# Pattern recognition receptor signaling and innate immune responses to schistosome infection

**DOI:** 10.3389/fcimb.2022.1040270

**Published:** 2022-10-21

**Authors:** Nouhoum Dibo, Xianshu Liu, Yunfeng Chang, Shuaiqin Huang, Xiang Wu

**Affiliations:** ^1^ Department of medical parasitology, Xiangya School of Basic Medicine, Central South University, Changsha, China; ^2^ Department of Forensic Medicine Science, Xiangya School of Basic Medicine, Central South University, Yueyang, China; ^3^ Hunan Provincial Key Lab of Immunology and Transmission Control on Schistosomiasis, Hunan Provincial Institute of Schistosomiasis Control, Yueyang, China

**Keywords:** schistosomiasis, innate immunity, pattern recognition receptor, cytokines, pathogenesis

## Abstract

Schistosomiasis remains to be a significant public health problem in tropical and subtropical regions. Despite remarkable progress that has been made in the control of the disease over the past decades, its elimination remains a daunting challenge in many countries. This disease is an inflammatory response-driven, and the positive outcome after infection depends on the regulation of immune responses that efficiently clear worms and allow protective immunity to develop. The innate immune responses play a critical role in host defense against schistosome infection and pathogenesis. Initial pro-inflammatory responses are essential for clearing invading parasites by promoting appropriate cell-mediated and humoral immunity. However, elevated and prolonged inflammatory responses against the eggs trapped in the host tissues contribute to disease progression. A better understanding of the molecular mechanisms of innate immune responses is important for developing effective therapies and vaccines. Here, we update the recent advances in the definitive host innate immune response to schistosome infection, especially highlighting the critical roles of pattern recognition receptors and cytokines. The considerations for further research are also provided.

## Introduction

Schistosomiasis remains to be a significant public health problem in tropical and subtropical regions. Infection takes place when the final host is exposed to infective cercariae of *Schistosoma* which actively penetrate through the skin and rapidly transform into schistosomula. *S. japonicum*, *S. mansoni* and *S. haematobium* are the main species causing disease in human. *S. japonicum* is transmitted by the *Oncomelania* snail and causes intestinal and hepatosplenic schistosomiasis in China, the Philippines, and Indonesia. *S. haematobium* is transmitted by the *Bulinus* snail and causes urogenital schistosomiasis in Africa and some Arabian Peninsula. *S. mansoni* is transmitted by the *Biomphalaria* snail and causes intestinal and hepatic disease in Africa, the Arabian Peninsula and Latin America (McManus et al., 2020).

The continuous efforts of the national schistosomiasis control programs as well as the World Health Organization (WHO) technical and financial supports have contributed to the elimination or reduction of the prevalence of this devastating disease in several countries (Nelwan, 2019; Wiegand et al., 2021). However, it is estimated that this neglected tropical disease is still endemic in 78 countries, with more than 200 million people infected and almost 800 million people at risk worldwide (Molehin, 2020). WHO’s recommended strategy for controlling schistosomiasis is mass drug administration using the only available drug, praziquantel (PZQ) (Teixeira de Melo et al., 2013; Nelwan, 2019). PZQ is effective against adult worms but has less efficacy against juvenile forms and cannot prevent reinfection, warranting the need of alternative drugs or effective vaccines to facilitate the achievement of sustainable development goals (SDGs) for controlling this condition (Onasanya et al., 2021).

Similarly, remarkable efforts have been made in schistosomiasis vaccine research (Ramos et al., 2009; Tallima et al., 2015; Ricciardi et al., 2018; Riveau et al., 2018; You et al., 2018). Some vaccines, such as 28-kDa glutathione S-transferase of *S. haematobium* (Sh28GST), *S. mansoni* fatty acid-binding protein (Sm-14), and tetraspanin 2 protein (TSP-2) of *S. mansoni* (Sm-TSP-2) are at clinical trials (McManus et al., 2020), but there is no available prophylactic vaccine or therapy.

A comprehensive understanding of the host innate and adaptive immune responses to the invading schistosomes is vitally important for the development of the drugs and vaccines (El Ridi et al., 2010; Hu et al., 2017; Castro et al., 2018; Li et al., 2020; Amaral et al., 2021). Investigations focused to explore the host adaptive immune responses to the invading schistosomes have been well addressed, however, the functions of the host innate immunity in host-parasite interaction have not been systematically and comprehensively summarized.

The innate immune system is a universal and an ancient form of host defense strategy against pathogenic infection (Beutler, 2004; Mountford and Trottein, 2004). It mediates the recognition of pathogen-associated molecular patterns (PAMPs) including pathogen lipopolysaccharide (LPS), peptidoglycan, DNA and RNA, and sets the stage for the adaptive immune response (Aksoy et al., 2005; Thomas et al., 2005; Venugopal et al., 2009). Here, we summarize the recent progress on the pattern recognition receptors (PRRs) signaling, and the innate immune responses during schistosomiasis in a mammalian host.

## Schistosome recognition by innate immune cells and signaling pathways

The immune cells express many kinds of receptors in order to detect pathogens. These receptors are key elements of the innate immune system. They are mainly expressed by antigen-presenting cells (APCs) such as dendritic cells (DCs) and macrophages, but they are also found in other immune and non-immune cells (van Riet et al., 2009; Magalhães et al., 2010; Xu et al., 2014; Kaisar et al., 2018). There are five families of PRRs: Toll-like receptors (TLRs), Nucleotide-binding oligomerization domain-like receptors (NLRs), C-type lectin receptors (CLRs), RIG-1 like receptors (RLRs) (Takeuchi and Akira, 2010), and Absent in melanoma 2 (AIM2)-like receptors (ALRs) (Wang et al., 2019). Three of these PRRs (TLRs, CLRs and NLRs) have been well characterized in schistosomiasis.

### TLR-dependent innate immune signaling

TLRs are transmembrane PRRs. They regulate immune responses through the recruitment of the downstream adapters, including myeloid differentiation primary response protein 88 (MyD88), TIR domain-containing adapter protein inducing IFN-β (TRIF) and TRIF-related adapter molecule (TRAM). Depending on their cellular localization and agonist, the TLRs fall into two groups: plasma membrane-anchored TLRs (TLR1, 2, 4, 5 and 6) and endosomal TLRs (TLR3, 7, 8 and 9). The plasma membrane-anchored TLRs mainly recognize microbial membrane components while the endosomal TLRs predominantly recognize microbial nucleic acids (Liu et al., 2017). After schistosome detected, TLRs recruit adapter molecules to relay signals to downstream molecules, which results in the activation of the transcription of nuclear factor (NF)-κB and mitogen-activated protein kinases (MAPKs) pathways (Aksoy et al., 2005; Thomas et al., 2005; Venugopal et al., 2009).

**Figure 1:**
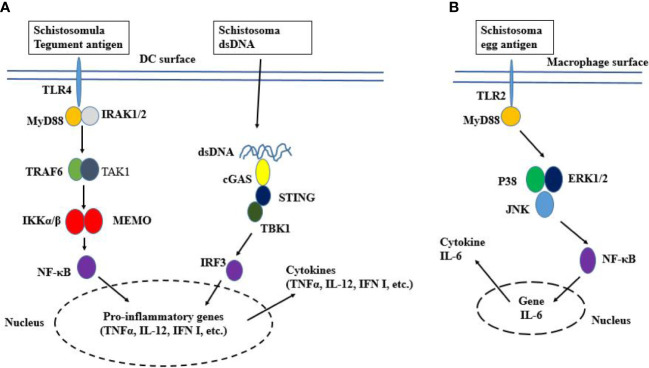
Schematic representation of TLR signaling. **(A)** TLR4 localizes to the cell surface. It is activated by ligand binding, which leads to the dimerization of TLR and the recruitment of TLR domain-containing adaptor proteins. Then MyD88 activates IRAK which induces K63-linked polyubiquitination on TRAF6 itself and TAK1. The TAK1 activation leads to the activation of IKK complex NF-κB and cytokine genes transcription. The worm’s DNA is sensed by cGAS, resulting in the activation of STING-TBK1-IRF3 signaling and IFN-I response. **(B)** Egg activated TLR2 recruits MyD88, resulting in the phosphorylation of P38, ERK1/2, and JNK. Thereafter, NF-κB induces the expression of the IL-6 gene.

Different antigens drive distinct immune responses *via* TLRs. The excretory-secretory (ES) products released within the first 3 h of cercarial transformation (termed 0-3 hRP) have been shown to induce IL-12p40 production in macrophages *via* TLR4-MyD88 pathway (Cardoso et al., 2008). Similarly, *S. mansoni* schistosomula tegument Sm29 antigen triggers TLR4-MyD88 pathway to induce the production of IL-12 and TNF-α in DCs (**Figure 1A**) leading to Th1 immune responses in murine schistosomiasis (Thomas et al., 2003; Durães et al., 2009). Also, the extracellular vesicles (EVs) released from the worm can activate macrophages-mediated immune signaling in murine schistosomiasis (Trottein et al., 2004; Jenkins et al., 2005). Molecular analysis revealed that the EVs released by schistosome contain microRNA (miRNA) that interacts with macrophages *via* TLR4 to regulate the expression of pro-inflammatory cytokines (Gong et al., 2018). However, another study revealed that the host’s miRNA is also involved in macrophages-mediated immune responses (Donnelly et al., 2010). In their study, Guiri and Cheng found that the host miR-148a targets the phosphatase and tensin homolog (PTEN) through PI3K/AKT pathway to regulate cytokine production in macrophages (Donnelly et al., 2010). As the worm can absorb the host product, it remains unclear whether the host miR-148a is released by the worm EVs or is directly produced by the macrophage. Further investigation are needed to resolve this issue. *via tensing*

The egg Lacto-N-fucopentose III (LNFPIII) triggers TLR4 to induce DC2 polarization and subsequent Th2 immune response (van Riet et al., 2009; Zhu et al., 2016). Besides, the dsRNA from an egg induces DC-driven Th2 immune responses *via* TLR3-MyD88 pathway (Aksoy et al., 2005). However, unknown ligands from eggs have induced type I IFN (IFN-I) production in DCs *via* the IFN-I receptor (IFNAR)-STAT-1 pathway (Liu et al., 2019), suggesting that IFN-I may be produced during schistosomiasis in TLR independent-manner. In addition, the soluble egg antigens (SEAs) can activate MyD88/NF-κB pathways of both TLR2 and TLR4 in murine macrophages. TLR4 signaling results in the production of TNF-α and IL-12 (Xu et al., 2014), whereas TLR2 signaling results in the production of IL-6 and monocyte chemoattractant protein-1 (MCP-1) (**Figure 1B**) (Tang et al., 2021). Interestingly, a relevant study revealed that the worm’s cysteine proteases can inhibit TLR3/4-TRIF mediated IFN-I production in macrophages, suggesting the complexity of the inflammatory response regulation by schistosomes (Giri and Cheng, 2019). *via fucopeptide*

In addition to professional APCs, TLRs have been functionally characterized on eosinophils and natural killer (NK) cells during schistosomiasis (Magalhães et al., 2010; Chen et al., 2019). Eosinophils function as effector cells and they may also play modulatory roles *via* cytokine production. *In vitro* study revealed that *S. mansoni* lipids extract such as lysophosphatidylcholine (LPC) and prostaglandin (PG)D2 can interact with human eosinophil expressing TLR2 to induce eicosanoid synthesis and inflammatory phenotype (Magalhães et al., 2010; Magalhães et al., 2018). It is worth mentioning that eicosanoids have complex functions, in some cases, they support inflammation such as enhancing pro-IL-1β biosynthesis, and in others cases, they block inflammatory processes, for instance, by destabilizing TNF-α transcript (Sheppe and Edelmann, 2021). NKs responds to the invading pathogens by exerting a direct cytotoxic effect or secreting various cytokines, particularly interferon-gamma (IFN-γ) (Li et al., 2015). Both TLR3 and TLR4 can mediate NKs cell activation during schistosomiasis (Qu et al., 2018; Chen et al., 2019).

### CLR-dependent innate immune signaling

While the TLRs represent the most extensively characterized family of PRRs during schistosomiasis, there is an increasing understanding of the role of CLRs in innate immune activation. CLRs belong to a large family of proteins that contain a carbohydrate recognition domain (CRD) that in most cases binds sugars by binding to calcium ion (Ca^2+^) (Geijtenbeek and Gringhuis, 2009). Following PAMPs binding, CLRs induce multiple signal transduction cascades through their own immunoreceptor tyrosine-based activation motifs (ITAMs) or interacting with ITAM-containing adaptor proteins such as FcRγ. These signaling cascades lead to the activation of the NF-κB family of transcriptional factors through a Syk and CARD9-dependent pathway. The activation of NF-κB results in the production of pro-inflammatory cytokines and chemokines, which in turn attract leucocytes to the site of pathogen invasion or tissue damage (Kingeter and Lin, 2012). In contrast to TLRs, the main function of CLRs is to internalize antigens for the degradation, and to enhance antigen processing and presentation on major histocompatibility complex (MHC) classes I and II (Geijtenbeek and Gringhuis, 2009).

Several glycan moieties that interact with the CLRs have been identified in schistosome SEAs and migrating schistosomula ES products (Nyame et al., 2003; Vázquez-Mendoza et al., 2013; Pham et al., 2020). In human schistosomiasis, *S. mansoni* glycolipid induces DC-driven Th1 immune responses by the cooperation of DC-specific intercellular adhesion molecule-3-grabbing nonintegrin (DC-SIGN) and TLR4 (van Stijn et al., 2010). The glycosylated ES products released by *S. mansoni* larvae can interact with both the mannose receptor (MR) and DC-SIGN to induce cytokine production in immune cells (Paveley et al., 2011; Kuipers and Nolte-'t Hoen, 2020). The *in vitro* investigation revealed that both SEAs and adult worm antigens can interact with the host macrophages through the specific ICAM-3 grabbing nonintegrin-related 1 (SIGNR1) (Saunders et al., 2009). In addition, human macrophage galactose-type C-lectin (MGL) can internalize *Schistosoma* glycan for processing and presentation (van Vliet et al., 2005). Relevant studies revealed that the glycoprotein IPSE/α1 and κ5 are the main components of eggs that interact with DCs through MR and SIGN to induce Th2 immune responses (van Die et al., 2003; Meevissen et al., 2012; Ponichtera and Stadecker, 2015).

Similarly, both Dectin-1 and Dectin-2 have been shown to interact with SEAs to induce eicosanoid PGE2 synthesis in DCs (Kaisar et al., 2018). PGE2 is known as the regulator of the activation, maturation, migration, and cytokine secretion of the innate immune cells (Agard et al., 2013). *In vitro* studies have shown that the liver/lymph node-specific SIGN (L-SIGN) can recognize multiply fucosylated fractions within an egg’s glycosphingolipids but cannot bind to glycosphingolipids from cercarial or adult schistosomal stages, probably due to the difference in structure (Meyer et al., 2007).

Recently, the Collectin Kidney 1 (CL-K1, encoded by COLEC11 on chromosome 2p25.3), a member of the vertebrate C-type lectin superfamily, has been identified as a PRR of the lectin complement pathway. An epidemiological study in Nigeria showed that high CL-K1 serum levels were associated with low risk of schistosome infection (Antony et al., 2015).

### NLR-dependent innate immune signaling

NLRs are major cytosolic PRRs, their involvement in the orchestration of innate immunity and host defense against pathogens often results in the cleavage of gasdermin and the release of IL-1β and IL-18. Beyond the inflammasome activation, they have also been involved in NF-κB and MAPK activation (Babamale and Chen, 2021).

**Figure 2:**
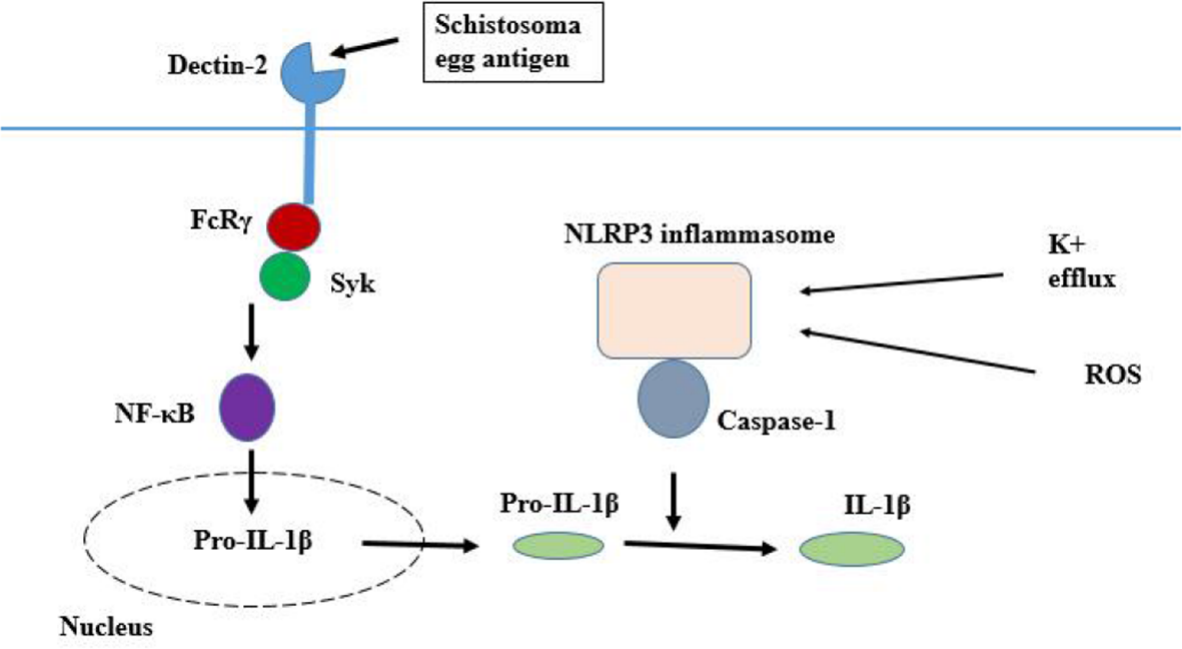
Inflammasome pathway. Egg antigen triggers the Dectin-2 receptor that couples with the FcRγ chain to activate the Syk kinase pathway, which controls the ROS and potassium efflux dependent manner, NLR inflammasome activation and IL-1β release.

NLR can be activated by a wide variety of PAMPs and danger-associated molecular patterns (DMPs), such as those of helminth (Celias et al., 2020), extracellular ATP, β-amyloid plaques, and uric acid crystals (Martinon et al., 2006; Kong et al., 2018). After the activation, NLRs recruit apoptosis-associated speck-like protein containing a caspase recruitment domain (ASC) and procaspase-1, and then oligomerize with them to form a functional inflammasome leading to the activation of caspase-1, which is responsible for the maturation of pro-inflammatory cytokines (Meng et al., 2016). SEAs can also trigger the Dectin-2 receptor that couples with the FcRγ chain to activate the Syk kinase pathway, which controls NLRP3 inflammasome activation and IL-1β release in reactive oxygen species (ROS) and potassium efflux dependent manner (**Figure 2**) (Ritter et al., 2010; Lu et al., 2017). A recent investigation revealed that NF-κB signaling is required for NLRP3 inflammasome activity (Zhang et al., 2019; Chen J et al., 2021). As a transcription factor, NF-κB probably mediates the transcription of pro-IL-1β and pro-IL-8, which are necessary for NLRP3 inflammasome activity (Chen et al., 2022). In addition to NLRP3 inflammasome, schistosome eggs can induce NLRP6 inflammasome activation in DCs, which also results in IL-1β production (Sanches et al., 2020). Most of the investigations carried out on NLR and inflammasome in schistosomiasis are focused on their role in an egg-induced immunopathology. Since the eggs’ antigens do not reflect the invading schistosome antigens, further investigation is still needed to identify the migrating schistosomula PMPs that may induce NLR inflammasome activation.

Recent works have revealed that the Cyclic GMP-AMP synthase (cGAS) is involved in *Schistosoma* DNA recognition and IFN-I production (Souza et al., 2020). When activated, cGAS catalyzes the formation of a second messenger, cyclic guanosine-adenosine monophosphate (cGAMP) which binds and activates the downstream Stimulator of Interferon Genes (STING) protein. STING then recruits Tank binding kinase 1 (TBK1) and activates the transcription factor interferon regulatory factor 3 (IRF3), ultimately leading to the production of IFN-I (**Figure 1A**) (Glück and Ablasser, 2019; Souza et al., 2020; Liang and Shen, 2022).

## Innate immune responses to the invading schistosome

The immediate pathogen response of innate immunity is achieved through the host PRRs that are located either in the cytosol or on multiple cellular surfaces, such as the plasma membrane and endosomal membrane. Following PAMPs recognition by PRRs, downstream signaling cascades including adaptors, kinases and transcription factors are activated *via* upstream signaling induction. These signaling pathways lead to the expression of anti-pathogen genes including pro-inflammatory cytokines, chemokines, and IFN-I to recruit immune cells to infected sites (Mogensen, 2009). In schistosomiasis, the host immune responses change throughout infection, dictated both by developmental changes in the parasite and tissue location. The intact adult worms are impervious to immune attacks, whereas developing skin-stage and lung-stage schistosomula are targets of protective immunity (El Ridi et al., 2010; Colley and Secor, 2014).

### Skin immune responses

In the skin, the cercariae induce the activation of keratinocytes and the complement system which promote the production of pro-inflammatory mediators including IL-1α, IL-1β, IL-33, thymic stromal lymphopoietin (TSLP), and leucocyte chemoattractant CCL20 (Hansell et al., 2008; Bourke et al., 2015). The cercaria glycocalyx is the main structure inducing the complement system activation and it is believed that its retention increases complement attack and subsequent immune responses (Da'dara and Krautz-Peterson, 2014). This preliminary immune response is followed by the activation of APCs and the migration of eosinophils, neutrophils and mast cells to the infected sites, which in turn display a direct cytotoxic effect or/and produce cytokines (Hogg et al., 2003; Kumkate et al., 2007; Paveley et al., 2009; McWilliam et al., 2013). However, the cytokines production profile differs according to the parasite species and the hosts. In the semi-permissive host, such as water buffalo, the skin immune response to *S. japonicum* is predominantly marked by the overproduction of IFN-γ, IL-4 and IL-10 (McWilliam et al., 2013). In human schistosomiasis*, S. mansoni* and *S. haematobium* up-regulate IL-1ra, IL-10, and TNF-α, whereas *S. japonicum* induces the up-regulation of several cytokines, including IL-1β, IL-1ra, IL-2, IL-6, IL-8, IL-10, IL-15, IL-18, and TNF-α (He et al., 2002; Winkel et al., 2018). In addition, the investigation on blood immune response in co-endemic areas for *S. mansoni* and *S. haematobium* revealed that the co-infected individuals release higher levels of the IL-10 than single species infected individuals or uninfected individuals (Turner et al., 2013; Meurs et al., 2014). However, there is no significant difference between the co-infected individuals and single species infected individuals in pro-inflammatory cytokines (IL-8 and TNF-α) production (Turner et al., 2013).

The skin physical barrier combined with effector molecule and cellular immune responses can effectively kill a number of invading *Schistosoma* larvae (Hogg et al., 2003; Freudenstein-Dan et al., 2003), but the parasites have a range of mechanisms to actively dampen the host’s immune response and promote their own survival (Sombetzki et al., 2018). More substantive data are available on the nature of schistosome molecules with immunoregulatory functions (Klaver et al., 2015; Angeles et al., 2020; Acharya et al., 2021).

### Lung immune responses

In contrast to the skin stage parasite, lung schistosomula elicit only a minimal inflammatory response. It is believed that this reduced immunogenicity is due to the change in the protein profile of the tegument, masking of parasite antigens *via* absorption of host protein and rapid tegumental turnover (Kusel et al., 2007; Burke et al., 2011). The host may also regulate inflammatory response at the lung stage for its survival because excessive inflammation can compromise pulmonary function (Burke et al., 2011).

Relevant studies revealed that the lung stage larvae of *S. mansoni* induced IL-4 production in rat (a semi-permissive host), whereas failed to induce the production of this cytokine in mice (a permissive host), suggesting that rat resistance to schistosomiasis is associated with Th2 cytokines (Badr et al., 2015). However, the lung stage larvae are able to induce Th1 and Th17 immune responses in mice, which play an important role in the parasite clearance (El Ridi et al., 2010). Another relevant study in *Microtus fortis* (a non-permissive host) revealed that the levels of several cytokines including IL-1β, IL-3, IL-4, IL-10, and IL-17 increased from the second to the third week post-infection (Hu et al., 2017). IL-1β has been shown to increase the level of plasma-free fatty acid and stimulate IL-1 and IL-12 mRNA expression, resulting in the reduction of the worm burden in BALB/c mice (El Ridi et al., 2006).

Lung immune responses to schistosomes involve nitric oxide (NO), which is mainly produced by macrophages (Liu et al., 2015; Shen and Lai, 2017). NO’s toxicity is linked to its ability to inhibit the parasite mitochondrial respiration (Shen and Lai, 2017). However, in avian schistosome infection (*Trichobilharzia regenti*), it was found that NO did not directly kill the worm but inhibited the activity of its cathepsins B1.1 and B2, the peptidases essential for the parasite migration and digestion (Macháček et al., 2016; Macháček et al., 2020).

## Role of innate immune responses in schistosomiasis pathology

The pathogenesis of schistosomiasis is associated with inflammatory responses directed against the eggs trapped in the host tissues (Liu et al., 2020). The eggs detection by the host PRRs induces signaling cascades leading to several DMPs generation and inflammation, which promotes the disease-associated pathology. Host regulators of liver fibrosis during human schistosomiasis have been reviewed previously (Kamdem et al., 2018), in this section, we highlighted the contribution of innate immune signaling in the disease-associated pathology.

Hepatic stellate cells (HSCs) activation is vital for liver fibrosis (Anthony et al., 2012). These cells can be activated by TLR4 in a cyclooxygenase 2 (COX2) and PGE2 axis-dependent manner during *S. japonicum* infection (Chen L et al., 2021). When activated, HSCs transdifferentiate into alpha-smooth muscle actin (α-SMA)-expressing myofibroblasts, which proliferate and secrete inflammatory cytokines and produce excessive extracellular matrix (ECM) resulting in liver fibrosis (Anthony et al., 2012; Chen L et al., 2021). Moreover, egg-induced TLR4 signaling promotes the expression of transglutaminase 2 (TGM2), an enzyme involved in ECM production (Wen et al., 2017). Although the mesenchymal stem cell (MSC) is being used to treat different immune-disturbance complications, current investigation revealed that TLR4 combined with IFN-γ can activate the MSC group with positive effects on the pathology of schistosomiasis by modulating Th subsets at some degree (Liu et al., 2020). In intestinal schistosomiasis, the granuloma-related cytokines production is mediated by TLR2 *via* the NF-κB pathway (Ashour et al., 2015). Interestingly, relevant studies revealed that the commensal bacteria can act as bystander activators of the intestinal innate immune system to instigate Th1 responses, suggesting the role of host microbiota in innate immune regulation (Holzscheiter et al., 2014).

The CLR CD209a has been shown to mediate IL-23 and IL-1β production in DCs, which drives Th17 dependent pathology in chronic schistosomiasis (Ponichtera et al., 2014; Ponichtera and Stadecker, 2015; Ponichtera and Stadecker, 2015). Similarly, both Dectin-2 and Mincle can induce IL-23 and IL-1β production in DCs *via* FcRγ-Syk signaling pathway, suggesting that DC-driven Th17 pathology involves multiple CLRs (Kalantari et al., 2018; Kalantari et al., 2019).

The role of NLRP3 inflammasome in *Schistosoma* egg-induced pathology has been extensively studied (Meng et al., 2016; Zhang et al., 2019). NLRP3 inflammasome mediates the conversion of IL-1β and IL-18 to their active form in response to pathogen infection. IL-1β and IL-18 influence adaptive immunity through modulation of Th cell subsets, skewing development in favor of Th1 and Th17 cells that are important in the pathogenesis. The *in vivo* experiment revealed that NLRP3 deficient mice infected with *S. mansoni* showed altered adaptive immune responses and decreased liver pathology (Ritter et al., 2010). In addition to inflammasome activation, SEAs can induce the production of TNF-α and TGF-β in Kupffer cells (KCs), these factors in turn promote ECM synthesis and liver pathology (Bieghs and Trautwein, 2013). Moreover, the TGF-β plays an important role in schistosomiasis-associated pulmonary arterial hypertension (PAH) (Kumar et al., 2015; Kumar et al., 2017). Kumar et al. reported that the type-2 inflammation driven by IL-4 and IL-13 promotes the TGF-β-induced PAH in murine schistosomiasis (Kumar et al., 2015; Kumar et al., 2019). Furthermore, the protein thrombospondin-1 (TSP-1) contributes to PAH *via* TGF-βpathway, and it has been demonstrated that the TSP-1 blockade can protect mice from *Schistosoma*-PAH (Kumar et al., 2017).

Current study revealed that *S. japonicum* egg antigens-induced ROS production in macrophages enhances macrophage polarization towards M2, which plays a critical role in liver pathology progression (Kamdem et al., 2018; Yu et al., 2021). In addition, the CCAAT/enhancer-binding homologous protein (CHOP), a transcriptional regulator induced by endoplasmic reticulum stress (ER stress), is also involved in M2-mediated pathology. A correlation between CHOP expression, STAT6 pathway activation and liver fibrosis has been observed in *S. japonicum* infected mice (Duan et al., 2019). Moreover, the interaction of *S. japonicum* SjE16.7 protein with the receptors for advanced glycation end products (RAGE) has been shown to promote colorectal cancer progression in NF-κB and ROS-dependent manner.

Although miRNAs are not widely studied for therapeutic use in pathogenic infection, there is an increasing understanding of their role in liver fibrosis regulation. The miRNAs improve fibrosis lesions by inhibiting the activation and proliferation of HSCs through the TGF-β. To date, several miRNAs exhibiting anti-fibrosis function in murine schistosomiasis have been characterized. Lentivirus-induced miRNAs (Let-7b) expression in mice alleviated liver fibrosis through the downregulation of TGF-β receptor I (TβRI) and the inhibition of Th1 and Th2 immune response (Tang et al., 2017). In addition, both miR-130a-3p and miR-200a can inhibit TGF-β dependent HSCs activation (Wang et al., 2017; Xu et al., 2021; Liu et al., 2021). Moreover, the mmu-miR-92a-2-5p has been shown to relieve liver fibrosis by regulating TLR2 signaling pathway (Zhao et al., 2019). In contrast to host miRNAs, the worm’s miRNAs promote liver fibrosis by inducing the expression of pro-inflammatory mediators during the chronic phase of infection (He et al., 2020; Wang et al., 2020).

## Harnessing innate immune signaling in vaccine research and schistosomiasis treatment

Recently, immunotherapies that can either activate or suppress innate immune responses are being investigated as treatment targets against schistosomiasis and the pathology they can cause. Current investigation revealed that PZQ plays an anti-splenomegaly role in chronic schistosomiasis by inhibiting the NLRP3 inflammasome activation in macrophages (Kong et al., 2018). In addition, PZQ has been shown to attenuate liver fibrosis by inhibiting the activation of HSCs and expression of collagen matrix in murine schistosomiasis (Liang et al., 2011; Liu J et al., 2019). However, in our opinion, the long-term complications of schistosomiasis in the individuals who recovered after PZQ treatment have not yet been clear and need more further investigations. Some therapeutic drugs such as boswellic acid-containing extract and genistein alleviate liver granuloma and fibrosis by regulating NF-κB signaling (Liu et al., 2014; Wan et al., 2017), and others, such as paeoniflorin by the inhibition of alternative macrophage activation *via* reducing the phosphorylation of JAK2 and STAT6 (Chu et al., 2011). Moreover, it has been reported that taurine exhibits anti-fibrotic function by inhibiting the thioredoxin-interacting protein (TXNIP)/NLRP3 inflammasome activities (Yu et al., 2016; Liu X et al., 2019).

With regard to vaccine development, some TLR agonists are under investigation for use as adjuvants to increase the effectiveness of the schistosomiasis vaccine. The glucopyranosyl lipid A (GLA) formulated in aluminum (GLA-Alum), a TLR4 ligand, combined with sm-p80 vaccine could induce balanced Th (Th1, Th2 and Th17) immune responses and significant sm-p80-specific antibodies production in vaccinated animals but did not maximize the immunogenicity and efficacity of the vaccine as expected (Zhang et al., 2018). Investigation on the ligands of TLR7/8 (R848) and TLR9 (CpG oligonucleotide) revealed that both of them block Treg immunosuppressive function and upregulate the production of TNF-α, IFN-γ, and several interleukins *in vitro* (Lu et al., 2013; Wang et al., 2013). Interestingly, *in vivo* experiment revealed that the combination of R848 and CpG with the DNA vaccine 26-kDa glutathione S-transferase of *S. japonicum* (pVAX1-Sj26GST) induces a significant protective immunity in animal model, suggesting that the combination of multiple TLR ligands may be targeted as a promising new approach for the design of *Schistosoma* vaccines (Wang et al., 2012; Wang et al., 2013).

## Challenges and perspectives for further research

Although numerous studies have been conducted to determine the role of the innate immunity in host-parasite interaction, its molecular mechanisms in schistosomiasis are not yet fully understood. This review provides an updated overview of the PRRs biology and innate immune signaling pathways. These innate immune receptors and their regulators-based intervention are potential targets to develop effective vaccine or inhibit the progression of chronic schistosomiasis. However, challenges remain in understanding of how do different activation pathways of PRRs converge or diverge, and how different innate immune signaling pathways are regulated at the molecular level. Priorities for further research include elucidating the signaling mechanisms involved in innate recognition of schistosomes and characterizing the active PAMPs from invading parasites that may successfully initiate innate immune responses and inflammatory signaling pathways. More efforts are also needed to clarify the immunomodulatory mechanisms of the TLRs agonists. Research on RNA sequencing and cytokines could also facilitate related studies.

## Author contributions

ND, SH and XW conceived the manuscript. ND and SH collected and reviewed the literature, and wrote the manuscript. ND and XL generated the figures and table. YC reviewed the literature. SH and XW reviewed and edited its final manuscript. All authors contributed to the article and approved the submitted version.

## Funding

The work was supported by the National Natural Science Foundation f China (82102428 to SH, 82072306 to XW), and the Natural Science Foundation of Hunan Province (2022JJ40663 to SH).

## Conflict of interest

The authors declare that the research was conducted in the absence of any commercial or financial relationships that could be construed as a potential conflict of interest.

## Publisher’s note

All claims expressed in this article are solely those of the authors and do not necessarily represent those of their affiliated organizations, or those of the publisher, the editors and the reviewers. Any product that may be evaluated in this article, or claim that may be made by its manufacturer, is not guaranteed or endorsed by the publisher.
